# Survey data on factors influencing participation in towel reuse programs

**DOI:** 10.1016/j.dib.2016.11.068

**Published:** 2016-11-24

**Authors:** Efthalia Dimara, Emmanouela Manganari, Dimitris Skuras

**Affiliations:** Department of Economics, University of Patras, Greece

## Abstract

This data article provides a data description on all subsamples related to the research article entitled “Don’t change my towels please: Factors influencing participation in towel reuse programs” (E. Dimara, E. Manganari, D. Skuras, 2017) [1]. The dataset was compiled from a field questionnaire survey of 1304 tourists in Greece that captured key demographic, behavioral and psychographic characteristics. The dataset is split into distinct sub-samples that differ depending on whether tourists chose to respond to certain parts of the questionnaire or not. We provide descriptive statistics for the entire sample and all sub-samples as well the questionnaire׳s version in English and the econometric procedures to analyze this complex dataset. This dataset will allow researchers to include the study׳s results in future elaborated meta-analyses and/or gain a better insight to the study׳s major conclusions.

**Specifications Table**

**Table:** 

Subject area	*Economics*
More specific subject area	*Consumer adoption of green hotel practices*
Type of data	*Tables and Text files*
How data was acquired	*Field Survey*
Data format	*Processed*
Experimental factors	*Random sampling of tourists on tourism hot spots in Greece*
Experimental features	*Sample selection of tourists with specific characteristics*
Data source location	*Greece*
Data accessibility	*Data is with this article*

**Value of the data**

• The data provide descriptive statistics for all sub-samples of the entire dataset and thus allow the inclusion of the research into future meta-analyses or meta-regressions with or without covariates.
• Researchers can gain a better insight into the study׳s design and results.
• Data and methods in this work can be valuable for the design and implementation of similar research in the future.

## 1 Data

In order to examine tourists’ willingness to participate in hotel towel reuse programs, and if yes, to estimate their willingness to pay and support financially the operation of such programs, a questionnaire survey was designed and implemented. The questionnaires were administered in tourism hot spots including Athens׳s Acropolis, ancient Olympia and the port of Patras during the summer months of 2014 using a direct face to face survey [1]. Appendix A provides a sample questionnaire in English administered to foreign tourists. The survey resulted to 1304 usable questionnaires, 689 completed by Greek and 615 by foreign tourists.

## 2 Experimental design, materials and methods

**Diagram 1:**
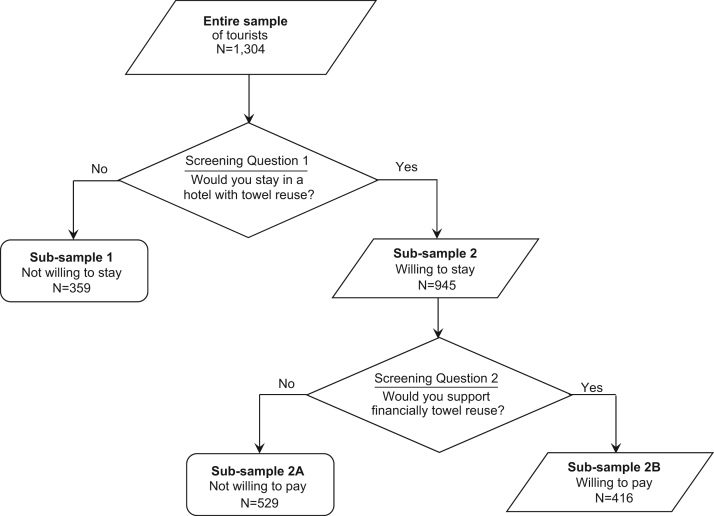
A flowchart of the questionnaire׳s screening questions and the resultant sub-samples.

A random sampling procedure of tourists was followed. The questionnaire addressed tourists who stayed in a hotel and knew the exact price of their accommodation and excluded all tourists from cruise ships or those staying at camp and caravan sites. The questionnaire contained two central screening questions that divided the entire sample into sub-samples according to their responses and preferences. Diagram 1 shows how the various sub-samples of the survey are generated. The first screening question concerned with the respondent׳s willingness to participate in a hypothetical towel reuse program, irrespective of her willingness to support financially the program. This splits the entire sample to sub-samples 1 and 2 in Diagram 1. Respondents not willing to participate in the program were asked to indicate the reasons why they prefer not to participate and the interview ended for them. Respondents willing to participate were the second screening question (Diagram 1) concerning with whether they would be willing to support this program financially or not. Responses to this question further split sub-sample 2 to those respondents who were not willing to pay (sub-sample 2A) but considered a towel reuse program worth having in a hotel, and to those willing to pay a positive amount of money (sub-sample 2B). Appendix B provides the descriptive statistics on a series of key demographic, socio-economic and psychographic characteristics for the entire sample and each one of the sub-samples depicted in Diagram 1.

Preferences to participate to a towel reuse program (screening question 1) are distinctively different between foreign and Greek tourists and between tourists who have a prior experience with a towel reuse program and those who have not. Appendix B also provides the descriptive statistics separately for Greek and foreign tourists and for tourists with or without prior experience of a towel reuse program. Finally, the analysis of the collected data demanded specific econometric processes. First, an analysis aiming to decompose the observed differences in the probability to respond “yes” to the first screening question between Greek and foreign tourists and between experienced and non-experienced with towel reuse tourists. This method is detailed in Appendix C. Second, the econometric methods for estimating the willingness to pay for those tourists wishing to participate to the towel reuse program (sub-sample 2) are somehow involved. This is due to two reasons. There is a considerable concentration of zero bids in the form of those tourists who wish to participate but are not willing to pay (sub-sample 2 A). At the same time sub-sample 2 has been selected out of the entire sample due to the “yes” response to the first screening question. Appendix C details the method for estimating a selection model with a notable concentration of zero responses.

## Footnotes

Appendix A Supplementary data associated with this article can be found in the online version at 10.1016/j.dib.2016.11.068.
