# Supplementary material for: Survey data on factors influencing participation in towel reuse programs
Source: Data Brief. 2016 Nov 24;10:26–9. doi: 10.1016/j.dib.2016.11.068 (PMC5137323; doi:10.1016/j.dib.2016.11.068)
Supplement: Supplementary material [file mmc2.docx]

**Appendix A**

**The Questionnaire – English version**

| *Questionnaire Number:* |  |  |  |
| --- | --- | --- | --- |

**«EMERGING RESEARCH ISSUES IN THE HOTEL INDUSTRY»**

The present research is conducted by the Department of Economics of the University of Patras. The research goal is to examine consumers’ attitudes toward green policies in the tourism sector.

It is anonymous and does not record any personal data.

“Towel reuse” in hotels is an environmentally friendly initiative. Customers have the option not to replace their towels every day, but instead every other day. This way, natural resources (i.e.; water, energy) are saved and pollution is reduced (e.g.; detergents, conditioners). At the same time, hotels reduce their operating expenses (washing costs, replacement and attrition of towels) by as much as 1 euro per double room.

Imagine that you have booked a hotel, which applies the “towel reuse” policy, and it’s up to you to choose between ***daily towel replacement and not daily towel replacement (i.e.; every other day towel replacement).***

Would you choose towel replacement every other day (instead of daily towel replacement)?

| Yes |  |  | No |  |
| --- | --- | --- | --- | --- |

***(if YES, continue to section A)***

1. **If not,** which is the most important reason for choosing daily towel replacement. (✓ Only one answer)

| Hygiene- Cleanliness. |  |
| --- | --- |
|  |  |
| I think that the hotel aims only at profitability. |  |
|  |  |
| I do not care about protecting the environment. |  |
|  |  |
| I do not think that this is a way for saving natural resources. |  |
|  |  |
| Other __________________________________________________________ |  |

***(Continue to section B)***

**A.**

2. If the hotel mandatorily applies the “towel reuse” initiative and given that the amount of money savings (around **1 euro** per double room for every day the towels are not replaced) is offered by the hotel to an environmental organization, would you be willing to pay an additional amount of money on top of the room rate and if so, how much?

| Yes |  |  | how much? _______ (*Euros per day of stay*) |  |
| --- | --- | --- | --- | --- |
|  |  |  |  |  |
| No ¨ι﷽﷽﷽﷽﷽﷽﷽﷽_____ μονής); τίου και αν να'.................................ς.................................................... |  |  |  | |

**If not,** choose one of the following reasons that you consider the most important (✓only one answer)

| I think that there is no need to pay an additional amount of money. |  |
| --- | --- |
|  |  |
| I have to pay less, considering that the hotel reduces its operational expenses. |  |
|  |  |
| Other ______________________________________ |  |

***Β.***

3. Gender:

| Male |  |  | Female |  |
| --- | --- | --- | --- | --- |

4. Age:

| 20 - 29 |  |
| --- | --- |
|  |  |
| 30 - 39 |  |
|  |  |
| 40 - 49 |  |
|  |  |
| 50 – 59 |  |
|  |  |
| 60 + |  |

5. Education:

| Middle/secondary School |  |
| --- | --- |
|  |  |
| High School or College |  |
|  |  |
| University Bachelor or higher / graduate level + |  |

6. Household size (*number of people who live under the same roof and accompany you on holidays*):

| 1 |  |  | 2 |  |  | 3 |  |  | 4 |  |  | 5+ |  |
| --- | --- | --- | --- | --- | --- | --- | --- | --- | --- | --- | --- | --- | --- |

7. Country of Permanent Residence: __________________________________

8. What is the average price of the room per day of the hotels you usually/typically book?

(price of double room)*?*

| 40 – 60 € |  |
| --- | --- |
|  |  |
| 61 – 80 € |  |
|  |  |
| 81 – 100 € |  |
|  |  |
| 101 – 150 € |  |
|  |  |
| 151 € or more |  |

9. What is the average duration of your stay in a hotel?

___________________ day(s)

10. Have you ever visited a hotel that applies the “towel reuse” initiative?

| Yes |  |  | No |  |
| --- | --- | --- | --- | --- |

11. **If yes**, did you participate in the initiative at least once (did you choose not to have your towels replaced for at least one day?)

| Yes |  |  | No |  |
| --- | --- | --- | --- | --- |

12. According to you, which is the standard/usual policy that hotels apply?

| Towel reuse |  |
| --- | --- |
|  |  |
| Daily towel replacement |  |

1. Which, in your opinion, is the most important reason, for the hotels to apply this initiative?

| Natural resources savings-environmental protection protection of the environment |  |
| --- | --- |
|  |  |
| Reduction of operational expenses |  |
|  |  |
| Other _______________________________________________________ |  |

1. In your daily life, do you try to save natural resources (e.g. paper recycling, use of energy saving bulbs, water saving practices, etc.)

| Never |  |
| --- | --- |
|  |  |
| Usually not |  |
|  |  |
| Usually yes |  |
|  |  |
| Always |  |

***Thank you for your participation!***
