# Supplementary material for: Survey data on factors influencing participation in towel reuse programs
Source: Data Brief. 2016 Nov 24;10:26–9. doi: 10.1016/j.dib.2016.11.068 (PMC5137323; doi:10.1016/j.dib.2016.11.068)
Supplement: Supplementary material [file mmc4.docx]

**Appendix C**

**Methods - Decomposing Probabilities and Modeling Willingness to Pay**

**C1. Probability Decomposition**

Tourists choose whether or not to participate in the offered towel reuse program by comparing the expected utilities of the two choices $U_{a}$ (to participate) and $U_{b}$ (to abstain). Tourists participate in the offered program if $U_{a}>U_{b}$, and this is recorded by screening question 1. As we do not observe or record all the attributes that affect their preferences we assume that a latent utility index model exists such that $y_{i}^{*}=U_{a}-U_{b}=\boldsymbol{z}_{\boldsymbol{i}}^{\boldsymbol{'}}\boldsymbol{a}+\varepsilon_{1,i}$ where $\boldsymbol{z}$ is a vector of the observed characteristics of tourists, $\boldsymbol{a}$ is a vector of the coefficients to be estimated, and $\varepsilon_{1}$is an error term $\varepsilon_{1}\sim N\left[ 0,\sigma_{1}^{2} \right]$. The outcome of screening question 1 is $\begin{matrix} y=1\text{ (tourist answers "yes" to screening question 1) if }y^{*}>0,\text{ i.e., }U_{a}>U_{b}\text{ and } \\ y=0\text{ (tourist answers "no" to screening question 1) if }y^{*}\leq0,\text{ i.e., }U_{b}>U_{a}\text{ } \end{matrix}$

This can is estimated through a probit model. Once the probit model is estimated for the sample of all tourists (entire sample), the mean sample towel reuse adoption rate can be estimated for the entire sample or for various sub-samples. For example, the adoption rate for Greek tourists is $\hat{y}^{G}=\bar{P}^{G}\left( \boldsymbol{z}^{\boldsymbol{'}}\boldsymbol{,}\hat{\boldsymbol{a}} \right)=\frac{1}{N^{G}}\sum_{i=1}^{N^{G}} \Phi\left( \boldsymbol{z}^{\boldsymbol{'}}\hat{\boldsymbol{a}} \right)$ where the function $\Phi\left( . \right)$ denotes the standard normal distribution, $\hat{y}^{G}$ and $\bar{P}^{G}\left( \boldsymbol{z}^{\boldsymbol{'}}\boldsymbol{,}\hat{\boldsymbol{a}} \right)$ are expressions of the average predicted rate and of the average probability of towel reuse adoption by Greek tourists and $N^{G}$ is the size of the sub-sample of those tourists. Correspondingly, the adoption rate for foreign tourists can be written as $\hat{y}^{F}=\bar{P}^{F}\left( \boldsymbol{z}^{\boldsymbol{'}}\boldsymbol{,}\hat{\boldsymbol{a}} \right)=\frac{1}{N^{F}}\sum_{i=1}^{N^{F}} \Phi\left( \boldsymbol{z}^{\boldsymbol{'}}\hat{\boldsymbol{a}} \right)$. The difference between average rates of adoption of the towel reuse program between the sub-samples of non-experienced and experienced tourist $\left( \hat{y}^{F}-\hat{y}^{G} \right)$ can be decomposed as $\hat{y}^{F}-\hat{y}^{G}=\left\{ \bar{P}\left( \boldsymbol{z}^{\boldsymbol{F}}\boldsymbol{,}\boldsymbol{a}^{\boldsymbol{F}} \right)-\bar{P}\left( \boldsymbol{z}^{\boldsymbol{F}}\boldsymbol{,}\boldsymbol{a}^{\boldsymbol{G}} \right) \right\}+\left\{ \bar{P}\left( \boldsymbol{z}^{\boldsymbol{F}}\boldsymbol{,}\boldsymbol{a}^{\boldsymbol{G}} \right)-\bar{P}\left( \boldsymbol{z}^{\boldsymbol{G}}\boldsymbol{,}\boldsymbol{a}^{\boldsymbol{G}} \right) \right\}$. where $\bar{P}\left( \boldsymbol{z}^{\boldsymbol{F}}\boldsymbol{,}\boldsymbol{a}^{\boldsymbol{G}} \right)$ is the average predicted rate of towel reuse adoption across foreign tourists, with characteristics captured by the vector $\boldsymbol{z}^{\boldsymbol{F}}$ but using the coefficients estimated by the sub-sample of Greek tourists $\boldsymbol{a}^{\boldsymbol{G}}$. The first term in braces describes the change in average adoption rates arising from the changing coefficients, whereas the second term describes the changes arising from the changing observable characteristics of the tourists in the two categories. Appendix B of this work provides descriptive statistics for the Greek-foreign tourists and for tourists having prior experience or not of a towel reuse program.

**C2. Modeling Willingness-to-Pay**

The Willingness to Pay (WTP) for participating in the towel reuse program cannot be observed for those tourists who do not want to subscribe to the program i.e., those answering “no” to screening question 1. At the same time, more than half of the tourists who answer “yes” to screening question 1, are not willing to support the program financially for various reasons (sub-sample 2A). As such, modeling WTP requires first to take into account that sub-sample 2 is a “selection” of the entire sample and second that sub-sample 2 consists of a high concentration of responses truncated to “zero”. As such, WTP is modeled using a tobit model under sample selection with lower tail censoring at zero and latent underlying regression ${{\mathrm{WTP}_{i}}^{*}\boldsymbol{=}\boldsymbol{x}_{\boldsymbol{i}}\boldsymbol{\beta}}^{\boldsymbol{'}}+\varepsilon_{2}$ $\mathrm{with} \varepsilon_{2}\sim N\left[ 0,\sigma_{2}^{2} \right]$, where $\boldsymbol{\beta}$ is a vector of parameters to be estimated by the model and $\boldsymbol{x}_{\boldsymbol{i}}$ is the vector of observable characteristics for respondent i in each sub-sample. The descriptive statistics of respondents in each sub-sample is provided in Appendix B of this work. The tobit formulation takes the form:

${\mathrm{WTP}_{i}}^{*}=\left\{ \begin{aligned} {\boldsymbol{x}_{\boldsymbol{i}}\boldsymbol{\beta}}^{\boldsymbol{'}}+\varepsilon_{2}, if {\boldsymbol{x}_{\boldsymbol{i}}\boldsymbol{\beta}}^{\boldsymbol{'}}+\varepsilon_{2}>0 and y=1 \text{(sub-sample 2B)} \\ 0, if {\boldsymbol{x}_{\boldsymbol{i}}\boldsymbol{\beta}}^{\boldsymbol{'}}+\varepsilon_{2}\leq0 and y=1 \text{(sub-sample 2A)} \\ \mathrm{unobserved}, if y=0 \text{(sub-sample 1)} \end{aligned} \right.$

The conditional and unconditional expectation of WTP, i.e. the predicted average WTP for tourists stating positive values only (sub-sample 2B), and for all respondents stating a value (sub-sample 2), zero or positive, can be computed once the sample selected tobit model has been estimated. Thus, fitting this model will allow the estimation of the parameters vector $\boldsymbol{\beta}$, and the effects of independent variables on WTP. If there is evidence that the error term $\varepsilon_{2}$ is not normally distributed, other parametric forms that may fit the data better, such as the Weibull distribution, may be tested.
